# Quality of life and mental health in adolescent users of oral contraceptives. Results from the nationwide, representative German Health Interview and Examination Survey for Children and Adolescents (KiGGS)

**DOI:** 10.1007/s11136-020-02456-y

**Published:** 2020-03-06

**Authors:** Sabina Klara Lewandowski, Gunnar Duttge, Thomas Meyer

**Affiliations:** 1 Klinik für Psychosomatische Medizin und Psychotherapie, Georg-August-Universität Göttingen, Göttingen, Germany; 2 Zentrum für Medizinrecht, Georg-August-Universität Göttingen, Göttingen, Germany; 3 Department of Psychosomatic Medicine and Psychotherapy, German Centre for Cardiovascular Research, University of Göttingen, Von-Siebold-Str. 5, Göttingen, Germany

**Keywords:** Oral contraceptive use, Hormonal contraception, Adolescents, Quality of life, Mental health, Well-being

## Abstract

**Objective:**

Using data from the nationwide, cross-sectional KiGGS (German Health Interview and Examination Survey for Children and Adolescents) study, we investigated whether hormonal contraception in adolescents aged 15 to 17 years was linked to health-related quality of life and mental health problems.

**Methods:**

Study participants had undergone standardized recordings of blood pressure and measurements of serum 25-hydroxyvitamin D [25(OH)D]. Quality of life was assessed by self- and parent-rated KINDL-R questionnaires, whereas mental health problems were screened by means of the Strengths and Difficulties Questionnaire (SDQ).

**Results:**

Self-rated quality of life was similar between users (*n* = 522) and non-users (*n* = 1173, 69.2%) of oral contraceptives (69.2 ± 11.2 vs. 69.2 ± 11.0, *p* = 0.943), as was the parent-rated version (72.9 ± 10.6 vs. 72.9 ± 10.5, *p* = 0.985). Likewise, no significant differences were observed between the two groups with respect to both self- (10.9 ± 4.4 vs. 10.8 ± 4.6, *p* = 0.732) and parent-rated SDQ scores (7.2 ± 4.8 vs. 7.0 ± 4.6. *p* = 0.390). However, serum 25(OH)D (59.5 ± 32.9 vs. 46.1 ± 28.0 nmol/L, *p* < 0.001) and mean arterial blood pressure (88.2 ± 7.4 vs. 86.5 ± 7.7 mmHg, *p* < 0.001) were significantly higher in users than in non-users. There was a trend towards a higher rate of psychotropic drug prescription in participants taking oral contraceptive pills as compared to those not receiving hormonal contraception (17.8% vs. 14.4%, *p* = 0.052). A series of linear regression models with either KINDL-R or SDQ as dependent variable confirmed that there were no associations between components of mental well-being and contraceptive drug use, irrespective of whether self- or parent-ratings were included in these models.

**Conclusions:**

In a large, representative sample of German adolescents, exposure to exogenous contraceptive hormones was associated with higher arterial blood pressure and serum 25(OH)D concentration, whereas hormonal contraception was not linked to health-related quality of life or mental well-being.

## Introduction

Worldwide, oral contraceptives are a popular choice among women of reproductive age to avoid unintended and mistimed pregnancies and to alleviate menstrual pain, premenstrual syndrome, abnormal uterine bleeding, and severe acne. Despite long-term experience with oral contraceptives since they were first introduced into the market in 1959, clinical studies on their influence on mental health and quality of life are still rare [1–4]. Particularly, in adolescents and young women, the impact of sexual steroids, especially progesterone and estradiol, on depression has been controversially discussed, and it is unclear whether the development of mood changes and/or mental health disorders are clinically relevant side effects of hormone-based contraceptives [5–7]. A large study including 6654 sexually active women aged 25–34 years conducted in the USA reported that hormonal contraception may reduce the levels of depressive symptoms among young women and thus may elicit a protective function [8]. Similar results were reported from a small, cross-sectional Norwegian study in adult women showing a decreased likelihood of a current mood disorder in users of combined contraceptive preparations, whereas, in contrast, users of progestin-only agents experienced a deleterious effect [9]. One small study in women aged 14–20 years found no significant changes in self-rated quality of life perception or depressive symptoms, despite the fact that both the number of days of menstrual bleeding and the use of analgetic medication to relieve menstrual pain were both reduced [10]. In a double-blind, randomized, placebo-controlled trial in 340 young, healthy Swedish women aged 18–35 years, Zethraeus et al. demonstrated that a first-choice levonorgestrel-containing drug administered for the treatment of premenstrual symptoms resulted in a statistically significant reduction in general well-being, when compared to a placebo, but no statistically significant effects on depressive symptoms [11].

However, a few important and well-conducted clinical studies suggested influences of hormone-based contraception on the development of depressive symptoms. A large cohort study conducted in Danish adolescents and young women demonstrated that the use of oral contraceptives was positively associated with higher frequencies of subsequent use of antidepressants and a first diagnosis of depression [12]. Recently, the same group also reported that the use of hormonal contraception was positively associated with suicide attempt and suicide, which was particularly observed in adolescent women who experienced the highest relative risk [13]. A similar relationship between the use of hormonal preparations and suicidal tendency was found in 27,067 women aged 20 years or older in data from the Korea National Health and Nutrition Examination Survey, KNHANES [14].

Given the growing evidence that oral contraceptive use may impact on depressive mood and result in higher suicide rates, we considered whether also health-related quality of life known to be reduced in depressed subjects is likewise related to hormonal contraception. Therefore, the purpose of this study was to examine the association of hormone-based contraceptives with self- and parent-rated quality of life and mental health problems in girls aged 15–17 years. To this end, we performed a post hoc analysis using data from the representative and nationwide German Health Interview and Examination Survey for Children and Adolescents (Kinder- und Jugendgesundheitssurvey, KiGGS) to determine whether those that use oral contraceptives differ from their non-medicated counterparts in terms of mental well-being.

## Methods

### Design of the KiGGS study

The KiGGS study was conducted from May 2003 to May 2006 by the Robert Koch-Institute with the aim of establishing a nationally representative sample to assess physical, psychological, and social health in German children and adolescents according to the WHO health definition. In the total, 17,641 study participants up to 17 years of age were surveyed which corresponded to a response rate of 66.6%. A two-stage probability sampling strategy was applied [15]. In the first stage, a sample of 167 municipalities was drawn according to the structure of the federal states and the sizes of the municipalities of the Federal Republic of Germany. In the second stage, at each sample point, a random sampling from local population registries in proportion to the age and gender structure of the nationwide child population was performed. The local study teams consisted of five trained members, led by a physician experienced in pediatrics. The survey comprised physical and medical examinations including a comprehensive psychometric assessment. The present analysis is based on 1695 female study participants, 15–17 years of age, for whom valid measurements from psychometric testing and data on oral contraceptive use were available.

### Compliance with ethical standards

From all study participants aged over 15 years and their accompanying parents, written informed consent was obtained prior to each interview and examination. The study was approved by the Institutional Review Board of the Charité Universitätsmedizin Berlin (Ethics Committee of the Virchow Hospital, Humboldt University) and the German Federal Office for the Protection of Data [15].

### Collection of data

Data collection comprised a computer-assisted personal interview, self-administered psychometric assessment, and standardized physical examinations. Socioeconomic status (SES) was classified into three groups (low, medium, and high) using the Winkler index, which employed information about parents’ education, occupational status, and net household income [16]. Based on information on their nationality, country of birth, and year of immigration of both parents, the study participants were classified as having or not having a family immigration background [17]. Anthropometric measurements of body weight and height were performed using a portable Harpenden stadiometer (Holtain Ltd., Crymych, UK) and a calibrated electronic scale (SECA, Birmingham, UK), respectively. From these data, body-mass index (BMI) was calculated as weight in kilograms divided by the square of height in meters. Systolic and diastolic arterial blood pressure recordings were obtained non-invasively from two independent sequential readings taken after 5 min of rest in a sitting position with the subject’s right arm on the desk, the forearm in supination, and the elbow at the level of the right atrium [18–20]. The Datascope Accutorr Plus sphygmomanometer equipped with a portable monitor was used to detect oscillometric pulsations in the brachial artery during cuff deflation [21, 22]. With regard to the circumference of the subject’s upper right arm, four different sizes of inflatable cuffs were used, as the cuff had to cover at least two-thirds of the length of the upper arm from the axilla to the antecubital fossa. Serum 25-hydroxyvitamin D [25(OH)D] concentrations were measured from venous blood samples using an automated direct LIAISON chemiluminescence immunoassay (CLIA, DiaSorin), as described [23, 24].

### Assessment of oral contraceptive use

The study physicians performed a standardized face-to-face, computer-assisted personal interview with each participant, in which detailed information on medical and psychiatric history was collected. The drug-use history covered both prescribed and non-prescribed over-the-counter (OTC) drugs which had been consumed during the last 7 days prior to the interview including oral contraceptive preparations. Adolescents were encouraged to add data on the use of medicines themselves. For the purpose of verification, parents and caregivers were asked in advance to bring prescriptions or original packaging to the local examination sites. Detailed information on psychotropic medication, such as the duration of use, was not available. All reported medications were divided into various groups according to the Anatomical Therapeutic Chemical (ATC) classification system, including psychotropic drugs.

### Psychometric assessment

The generic, German-language KINDL-R (Kinder-Lebensqualitätsfragebogen) questionnaire was employed in the KiGGS study as a screening instrument for health-related quality of life [25, 26]. This questionnaire distinguishes six dimensions of health-related quality of life with reference to the past week: physical well-being, emotional well-being, self-esteem, family, friends, and every day functioning. It contains 4 items per each subscale which were measured on a 5-point Likert scale (never, seldom, sometimes, often, all the time). The subscale scores were transformed to a range of 0 to 100, with a higher total sum score representing better health-related quality of life. The second instrument used to estimate prevalence rates for psychopathological problems with respect to oral contraceptive use was the Strengths and Difficulties Questionnaire (SDQ). This well-evaluated psychometric screening tool for children and adolescents contains five different subscales measuring emotional symptoms, conduct problems, hyperactivity-inattention, peer relationship problems, and prosocial behavior [27–29]. Each of the 25 items of the SDQ is scored on a 3-point Likert scale with 0 = not true, 1 = somewhat true, or 2 = certainly true, with higher scores indicating greater problems, except for prosocial behavior, where a higher score indicates more positive behavior. A total difficulties score ranging from 0 to 40 was obtained by summing the scores of all subscales, except prosocial behavior.

### Statistical analyses

Anonymized data obtained from the Robert Koch-Institute were analyzed using the Statistical Package for the Social Sciences (SPSS, IBM, New York, USA) software, version 22. To ensure that the survey cohort was representative of the national population, sampling weights were included in the analysis, which accounted for unequal sampling probabilities by adjusting for deviations in demographic characteristics (age, sex, residence in West or East Germany, and level of urbanicity). Descriptive statistics for users and non-users of oral contraceptives were expressed as means and standard deviations or as frequencies and percentages in the case of categorical data. Significant differences between the two groups were tested using Student’s *t* test for numerical data and Chi-square test for categorical variables, respectively. Fisher’s exact probability test was performed to test whether psychotropic drug use was more common in hormonal contraceptive users. Pearson’s correlation test was used to assess relationships between blood pressure and dimensions of mental well-being. In order to assess the relationships between quality of life and psychopathological problems, respectively, and oral contraceptive use, a series of linear regression models were calculated using components of mental well-being (self- and parent-rated KINDL-R or SDQ sum score) as the dependent variable and hormonal contraception as independent variable. Based on the univariate comparisons, these models were adjusted for age, body-mass index, migration status, log-transformed values for 25(OH)D, and mean arterial blood pressure. In addition, psychotropic medication use was added to the list of confounders, since from the existing literature on adolescents, it is known that hormonal contraception increases the risk of psychotropic drug use [30]. Migration status was included as a confounder in these models, since cultural diversity is known to influence contraceptive use. Furthermore, we computed mediation models using the SPSS macro PROCESS v2.10 [31] to test for possible interaction effects of (1) serum 25(OH)D on the relationship between oral contraceptive use and mean arterial blood pressure and (2) of blood pressure mediating indirect effects of oral conception on serum 25(OH)D. In order to keep the results comparable to the linear regression models, the covariates entered into the mediation models were kept the same. The mediator variable in the models was considered to be significant, when the 95th percentile-based confidence interval for the indirect effect did not exceed zero. In all tests, statistical significance was defined as *p* < 0.05.

## Results

### Sociodemographic characterization of the study population

Approximately one-third of all female adolescents aged 15–17 years (*n* = 522, 30.8%) reported current medication with oral contraceptive pills. The percentage of study participants exposed to hormonal contraceptives increased steadily with age, as only 85 out of 560 girls (15.2%) in the youngest age stratum of 15 years were taking these drugs as compared to 184 out of 571 (32.2%) participants who were 16 years old. Among the age group of 17-year-old girls, nearly half used hormonal forms of contraception (254 out of 564, 45.0%). Study participants taking hormonal contraceptives were older as compared to non-users (16.8 ± 0.8 vs. 16.3 ± 0.9 years, *p* < 0.001) and had a non-significantly higher body-mass index (22.4 ± 3.5 vs. 22.2 ± 4.3 kg/m^2^, *p* = 0.327). Participants with an immigration background were less likely to be exposed to hormonal contraceptives (6.7% vs. 20.7%, *p* < 0.001). However, the two groups did not differ with respect to their SES (*p* = 0.304). A detailed epidemiological characterization of the total study cohort dichotomized along the use of oral contraceptives is given in Table 1.

**Table 1:** Descriptive statistics of the total study cohort and comparison between the two groups of female KiGGS study participants aged 15–17 years with and without oral contraceptive use

	Total study population (*n* = 1695)	Participants using hormonal contraception (*n* = 522)	Participants not using hormonal contraception (*n* = 1173)	*p*-value
Age (years)	16.5 ± 0.9	16.8 ± 0.8	16.3 ± 0.9	< 0.001
Migrants (%)	16.4	6.7	20.7	< 0.001
Body-mass index (kg/m^2^)	22.3 ± 4.0	22.4 ± 3.5	22.2 ± 4.3	0.327
Socioeconomic status (%)				
Low	25.9	24.8	26.4	0.304
Medium	46.9	49.7	45.6	
High	27.2	25.5	27.9	
Systolic blood pressure (mmHg)	114.4 ± 9.4	115.6 ± 8.9	113.9 ± 9.5	< 0.001
Mean blood pressure (mmHg)	87.0 ± 7.7	88.2 ± 7.4	86.5 ± 7.7	< 0.001
Diastolic blood pressure (mmHg)	69.5 ± 7.1	70.6 ± 6.9	69.0 ± 7.2	< 0.001
Serum 25-hydroxyvitamin D (nmol/L)	50.2 ± 30.3	59.5 ± 32.9	46.1 ± 28.0	< 0.001

*KINDL-R* Children’s Quality of Life Questionnaire (Revidierter Fragebogen für KINDer und Jugendliche zur Erfassung der gesundheitsbezogenen Lebensqualität), *SDQ* Strengths and Difficulties Questionnaire

### Blood pressure and serum vitamin D in users and non-users

**Fig. 1:**
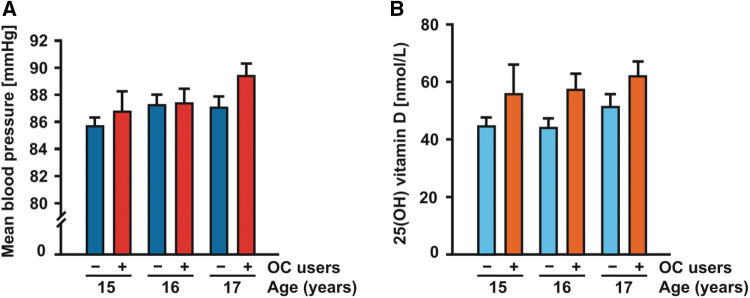
Histograms showing the means and standard errors of mean arterial blood pressure recordings (**a**) and serum 25-hydroxyvitamin D [25(OH)D] levels (**b**) by age groups in oral contraceptive (OC) users (red columns, marked with “ + ”) and non-users (blue columns, marked with “ − “). (Color figure online)

Since previous work from our and other groups has shown that, in adolescent KiGGS study participants, higher systolic blood pressure recordings and elevated vitamin D levels are both linked to better well-being and low distress [20, 32, 33], we next investigated the relationship between hormonal contraception and these two somatic parameters. As demonstrated in Table 1, both systolic (115.6 ± 8.9 vs. 113.9 ± 9.5 mmHg, *p* < 0.001) and diastolic blood pressure recordings (70.6 ± 6.9 vs. 69.0 ± 7.2 mmHg, *p* < 0.001) were higher in young women using oral contraception as compared to non-users. In study participants, who took oral contraceptives, the calculated mean arterial blood pressure was on average 1.7 mmHg higher than in non-users (88.2 ± 7.4 vs. 86.5 ± 7.7 mmHg, *p* < 0.001). In addition, serum 25(OH)D levels were significantly higher in oral contraceptive users than in non-users (59.5 ± 32.9 vs. 46.1 ± 28.0 nmol/L, *p* < 0.001). Figure 1 shows the mean blood pressure (A) and serum 25(OH)D levels (B) by age groups separately for the users and non-users of hormonal contraceptives.

### Mental health in users and non-users of hormonal contraceptives

Univariate comparisons were performed to address whether the hormonal contraception status was linked to changes in health-related quality of life and mental health. Our data demonstrated that the self-rated KINDL-R scores did not differ between the two groups of current users and non-users of oral contraceptives (69.2 ± 11.2 vs. 69.2 ± 11.0, *p* = 0.943, Table 2). Although the results from self-rating and proxy-rating by parents correlated significantly with each other (*r* = 0.533, *p* < 0.001), parents more often reported higher KINDL-R scores than their adolescent daughters. Again, there was no difference in parent-rated KINDL-R scores between users and non-users (72.9 ± 10.6 vs. 72.9 ± 10.5, *p* = 0.985). The hormone group and the non-hormone group did not significantly differ with respect to self- (10.9 ± 4.4 vs. 10.8 ± 4.6, *p* = 0.732) and parent-rated SDQ (7.2 ± 4.8 vs. 7.0 ± 4.6, *p* = 0.390). Similar to the KINDL-R data, the self- and proxy-rated SDQ versions correlated highly with each other (*r* = 0.483, *p* < 0.001). Next, we tested whether psychotropic drug prescription was more frequent in participants taking oral contraceptive pills compared with those not using hormonal contraception. In total, 93 participants using oral contraceptives reported additional psychotropic drug medication (17.8%), while only 14.4%, namely 169 out of 1173 non-users were taking psychotropic drugs (Table 2). In contrast, experience with psychotherapeutic treatment was more frequent in non-users (1.4%) as compared to users (1.0%) of oral contraceptives, although this difference was not at all statistically significant (*p* = 0.478). In summary, in univariate analysis, there was a trend towards a higher rate of psychotropic drug prescription in girls taking oral contraceptive pills compared with those not using hormonal contraception (*p* = 0.052), but psychometric indicators for mental well-being were similar in the two groups.

**Table 2:** Parameters of health-related quality of life and mental health in adolescent users and non-users of oral contraceptives

	Total study population (*n* = 1695)	Participants using oral contraception (*n* = 522)	Participants not using contraception (*n* = 1173)	*P*-value
Self-rated KINDL-R	69.2 ± 11.0	69.2 ± 11.2	69.2 ± 11.0	0.943
Parent-rated KINDL-R	72.9 ± 10.5	72.9 ± 10.6	72.9 ± 10.5	0.985
Self-rated SDQ	10.8 ± 4.6	10.9 ± 4.4	10.8 ± 4.6	0.732
Parent-rated SDQ	7.1 ± 4.7	7.2 ± 4.8	7.0 ± 4.6	0.390
Psychotropic medication (%)	15.5	17.8	14.4	0.052
Psychotherapeutic treatment (%)	1.3	1.0	1.4	0.478

### Somatic and psychosocial parameters associated with hormonal contraception

A series of linear regression models was performed with either self- or parent-rated KINDL-R or SDQ as the dependent variable and contraceptive drug use as independent variable to test for associations with blood pressure and 25(OH)D (Table 3). Notably, oral contraceptive use failed to be significantly associated with all parameters of mental well-being, irrespective of whether self- or parent-ratings were included in these models. However, in all four models psychotropic medication was a significant and inversely associated predictor of mental well-being (in all models, *p* < 0.001). In three of the four models tested, blood pressure and serum 25(OH)D were independently and significantly linked to mental well-being (Table 3). While blood pressure was significantly linked to self-rated KINDL-R and both self- and proxy-rated SDQ (in all models, *p* ≤ 0.001), it failed to reach significance when parent-rated KINDL-R was used as the dependent variable (*p* = 0.326, Model 2 in Table 3). In all models, higher circulating serum vitamin D concentrations significantly predicted better mental well-being, except for self-rated SDQ, where this relationship was observed only as a trend (*p* = 0.075).

**Table 3:** Results from a series of linear regression models with the indicated components of mental well-being (self- and patent-rated KINDL-R and SDQ, respectively) as the dependent variable and hormonal contraception as independent variables, adjusted for age, body-mass index, migration status, psychotropic medication use, serum 25-hydroxyvitamin D, and mean arterial blood pressure

	Model 1 Dependent variable: self-rated KINDL-R (*R*^2^ = 0.046, *p* < 0.001)			Model 2 Dependent variable: parent-rated KINDL-R (*R*^2^ = 0.040, *p* < 0.001)			Model 3 Dependent variable: self-rated SDQ (*R*^2^ = 0.065, *p* < 0.001)			Model 4 Dependent variable: parent-rated SDQ (*R*^2^ = 0.071, *p* < 0.001)		
	*B*	95% CI	*P*-value	*B*	95% CI	*P*-value	*B*	95% CI	*p*-value	B	95%-CI	*p*-value
Age (per year)	− 0.014	− 0.816 to 0.788	0.973	0.256	− 0.530 to 1.041	0.523	− 0.297	− 0.627 to 0.034	0.078	− 0.293	− 0.627 to 0.042	0.087
Body-mass index (kg/m^2^)	− 0.138	− 0.303 to 0.027	0.101	− 0.239	− 0.400 to − 0.078	0.004	0.154	0.086 to 0.222	< 0.001	0.175	0.106 to 0.244	< 0.001
Migration status	1.663	− 0.160 to 3.485	0.074	0.631	− 1.237 to 2.498	0.508	− 1.237	− 1.986 to − 0.488	0.001	− 1.241	− 2.013 to − 0.468	0.002
Psychotropic medication use	− 4.341	− 6.230 to − 2.452	< 0.001	− 4.853	− 6.710 to − 2.997	< 0.001	1.484	0.706 to 2.263	< 0.001	1.725	0.928 to 2.521	< 0.001
Serum 25-hydroxyvitamin D	2.418	0.044 to 4.793	0.046	2.571	0.249 to 4.893	0.030	− 0.892	− 1.873 to 0.089	0.075	− 1.506	− 2.498 to − 0.514	0.003
Arterial blood pressure (mmHg)	0.197	0.105 to 0.288	< 0.001	0.054	− 0.035 to 0.143	0.326	− 0.085	− 0.122 to − 0.047	< 0.001	− 0.064	− 0.102 to − 0.026	0.001
Hormonal contraception	− 0.145	− 1.656 to 1.366	0.851	0.038	− 1.429 to 1.506	0.959	0.312	− 0.311 to 0.935	0.326	0.577	− 0.053 to 1.206	0.073

Using a mediation model (Model 1 in Table 4), we confirmed the direct effect of oral contraceptive use on mean arterial blood pressure (*p* = 0.021), but did not find any indication of serum 25(OH)D being a significant mediator of this relationship (95% CI =  − 0.254 to 0.094). Similarly, as shown in Model 2 in Table 4, oral contraception was positively associated with serum 25(OH)D levels (*p* < 0.001), whereas blood pressure did not elicit any significant interacting effect on this relationship (95% CI =  − 0.007 to 0.001).

**Table 4:** Results from two mediation models to test for possible interaction effects of 25-hydroxyvitamin D [25(OH)D] on the relation between oral contraceptive use and mean arterial blood pressure (Model 1) or blood pressure on the relation between oral contraceptive use and 25-hydroxyvitamin D (Model 2), demonstrating the bootstrapped estimates, 95% confidence intervals (CIs), and the corresponding *p* values

	Model 1 Outcome: mean arterial blood pressure (*R*^2^ = 0.056, *p* < 0.001)			Model 2 Outcome: serum 25(OH)D (*R*^2^ = 0.071, *p* < 0.001)		
	Coefficient	95% CI	*p*-value	Coefficient	95% CI	*p*-value
Oral contraception	1.324	0.198 to 2.450	0.021	0.084	0.043 to 0.124	< 0.001
Age	1.146	0.551 to 1.741	< 0.001	0.034	0.012 to 0.056	0.002
Body-mass index	0.252	0.128 to 0.377	< 0.001	− 0.009	− 0.013 to − 0.004	< 0.001
Migration status	0.893	− 0.544 to 2.330	0.223	0.089	0.037 to 0.141	0.001
Psychotropic medication use	0.134	− 1.696 to 1.965	0.886	− 0.027	− 0.094 to 0.040	0.425
Serum 25(OH)D (Model 1) or mean arterial blood pressure (Model 2)	− 0.942	− 2.774 to 0.889	0.313	− 0.001	− 0.004 to 0.001	0.313
Indirect effects of contraception on mean arterial blood pressure				Indirect effects of contraception on serum 25(OH)D		
Serum 25(OH)D	Effect: − 0.077	95% CI − 0.254 to 0.094		Mean blood pressure Effect: − 0.002	95% CI − 0.007 to 0.001	

## Discussion

This post hoc analysis was performed to test the clinically relevant hypothesis that female adolescents exposed to oral contraceptives had a reduced health-related quality of life and more psychopathological problems, which may precede the elevated suicidal tendency and increase in the number of suicide attempts recently reported in the literature for users of these drugs. Using data from the nationwide, representative KiGGS study, we found, however, that oral contraceptive use in German girls was unrelated to two well-established and independent psychometric measurements for mental well-being, namely health-related quality of life as measured by the KINDL-R and psychological distress and behavioral problems as measured by the SDQ. Neither KINDL-R nor SDQ scores were linked to the contraception status, regardless whether univariate comparisons or adjusted regression models were performed. The lack of a significant relationship between subjective parameters of mental well-being and hormonal contraception was independent of whether data from self- or parent-rated assessment were analyzed. In contrast, oral contraception was positively linked to a higher probability of taking psychotropic medication, in both univariate and multivariate analyses. Additionally, we observed that two somatic parameters, namely arterial blood pressure and serum 25(OH)D concentration, were elevated in users of oral contraceptives compared to non-users. Notably, these positive associations were observed in three out of four regression models calculated, whereas there was no evidence that blood pressure mediated indirect effects of oral contraception on serum 25(OH)D and vice versa, suggesting that the two factors functioned independently in the context of hormonal contraception.

Our data from the KiGGS study confirm previous results showing that hormonal contraception, especially among adolescents, was linked to a higher rate of subsequent use of antidepressants [12]. There is evidence that daily ratings of mood and sexual interest follow hormonal changes during the menstrual cycle, since depression scores are highest during the premenstrual phase and lowest during the postmenstrual phase, whereas sexual interest peaked in the postmenstrual phase and bottomed out in the premenstrual phase [34]. Unfortunately, however, information on the actual state of the menstrual cycle at the time of the study interview was not available in the KiGGS public use file.

Sex hormones act through binding to their specific nuclear receptors and are imported as transcriptionally active complexes into the nucleus to modulate gene induction in a ligand-dependent manner. Estrogen and progesterone receptors are widely expressed in the central nervous system, where they execute multiple and diverse non-reproductive functions. It is conceivable that the different steroid hormones and their metabolites affect brain excitability in a complex and regulated manner, as well as orchestrating menstrual cyclicity [11].

In previous work using data from a subsample of the KiGGS study participants, our group as well as others demonstrated that low serum 25(OH)D levels were linked to more emotional and behavioral problems [32, 33]. Likewise, in Iranian boys and girls with vitamin D-insufficiency or -deficiency (≤ 30 ng/mL), the prevalence of self-reported sadness or depression and poor sleep quality was significantly higher than in school students with normal vitamin D levels [35]. An Italian study in 158 young women, aged 15–21 years, with premenstrual syndrome reported that vitamin D supplementation over 4 months can be regarded as an effective and convenient method for improving both quality of life and the intensity of mood disorders associated with severe hypovitaminosis D and symptoms of premenstrual syndrome [36]. In a prospective, population-based birth cohort from South West England, Tolpannen et al. demonstrated that higher concentrations of 25(OH)D_3_ assessed at a mean age of 9.8 years were associated with lower levels of depressive symptoms at age 13.8 years [37].

In a prospective, population-based Canada-wide observational study, Brajic et al. demonstrated less hip region peak bone mineral density (BMD) accrual in adolescent users of combined hormonal contraceptives than in non-users [38]. This finding confirms previous studies demonstrating that the physiological BMD increase is lower in adolescents with long-term use of birth-control pills than in non-users [39–41]. Since the main effect of active vitamin D metabolites is to stimulate the absorption of calcium from the gut resulting in the mineralization of organic bone matrix, our finding of elevated vitamin D levels under hormonal contraception may not be fully unexpected. Given the positive correlation between serum vitamin D and quality of life, our observation linking oral contraceptive use to elevated vitamin D concentrations may be regarded as a compensatory mechanism which protects against a decline in mental health.

Similarly, elevations in blood pressure may account for the lack of decline in subjectively measured distress and mental well-being, as was observed here in users of hormonal contraceptives. Numerous studies have shown that long-term administration of oral contraceptives induced a mild elevation of systemic blood pressure also in late adolescence [42, 43]. In a cross-sectional analysis on 1248 adolescents aged 17 years, the investigators of the Western Australian Pregnancy (Raine) study found that girls using oral contraceptives had 3.3 and 1.7 mmHg higher systolic and diastolic blood pressure, respectively, compared with non-users [43]. In an important paper, Du et al. demonstrated that KiGGS study participants using hormonal contraceptives have a more unfavorable profile of cardiovascular risk factors and adverse health behaviors [44]. The drug-related slight increase in systemic blood pressure may elicit an overt stress-dampening effect, which in users of oral contraceptives prevents a relevant decline in mental well-being probably by suppressing negative mood or masking depressive symptoms.

These findings should be interpreted in the light of several inherent limitations which resulted mainly from the cross-sectional study design, prohibiting any causal interpretation or conclusions on a temporal relationship. Data for this study were neither collected by the investigators for the purpose of this novel research question nor were data collection methods predetermined and controlled for complete and valid information on potential confounding factors. The public use file did not specify the nature or indication of the contraceptive and psychotropic drug medication and, in particular, no information was available on the distribution of antidepressants in current users and non-users of oral contraceptives. Du and co-authors reported that, in KiGGS participants, the most frequently used oral contraceptives (> 90%) were single-phase combined preparations with a fixed amount of ethinyl estradiol and progestin, with nearly one-half of users taking these drugs for a minimum of 1 year [44]. Furthermore, there were no data on the season of study enrollment or on direct measurements of sunlight exposure known to affect dermal synthesis of vitamin D. An additional weakness of this post hoc study is that, due to the sample heterogeneity, the reported effect sizes were generally very small. However, the large sample size and the sophisticated method of sampling based on a random selection strategy allowed the detection of even weak associations which may be functionally relevant and should be verified in future studies using independent samples.

In summary, this large observational study found no evidence that the use of oral contraceptives in late adolescence is associated with a decline in health-related quality of life or mental well-being. This lack of a significant relationship between drug intake and mental health in this young cohort may be explained by the presence of compensatory pathways, and among them, serum vitamin D and blood pressure are interesting candidates for further investigations.

## Abbreviations

BMI: Body-mass index
KiGGS: German Health Interview and Examination Survey for Children and Adolescents (Kinder- und Jugendgesundheitssurvey)
KINDL-R: Children’s Quality of Life Questionnaire (Kinder-Lebensqualitätsfragebogen)
25(OH)D: 25-Hydroxyvitamin D
SDQ: Strengths and Difficulties Questionnaire
